# Beneath the numbers: a qualitative study of the politics of resistance to transparent workforce data reporting

**DOI:** 10.1108/JHOM-02-2025-0114

**Published:** 2025-09-26

**Authors:** Pauline A. Nelson, Fay Bradley, Abigail Tazzyman, Jane Ferguson, James Higgerson, Jon Gibson, Damian Hodgson

**Affiliations:** Sheffield University Management School, The University of Sheffield, Sheffield, UK; School of Health Sciences, The University of Manchester, Manchester, UK; Sheffield Methods Institute, The University of Sheffield, Sheffield, UK; Health Services Management Centre, University of Birmingham, Birmingham, UK

**Keywords:** Calculative practices, Transparency, Reactivity, Management-clinician relations, Workforce data, Metrics, Healthcare

## Abstract

**Purpose:**

The purpose of this paper is to go beyond assumptions of “objective” transparency in data reporting within healthcare systems by examining forms of resistance among general practice providers to sharing workforce data and managers’ interpretations of, and responses to, these resistances. Framing data reporting as a “calculative practice”, the article analyses the neglected political and/or performative dimensions of data to examine contested meanings and motivations, reflecting power struggles with implications for wider health and public sector policy.

**Design/methodology/approach:**

Drawing on qualitative individual and group interviews with 56 general practitioner service providers and managers (commissioners and/or policymakers), using thematic template analysis informed by the literature on reactivity effects, the article explores the nature and implications of resistances in this data reporting practice.

**Findings:**

Felt but hidden tensions between stakeholders involving distinct forms of resistance to workforce data reporting (the calculative practice) were revealed. These “surface”, “deeper” and “root” layers of resistance saw providers covertly contest the practice, which some believed was designed to scrutinise business models. Solutions presented to address resistance often misdiagnosed the nature of providers’ concerns by focusing on less political sources of resistance and neglecting root challenges of power and trust.

**Originality/value:**

The article highlights the value of political rather than rationalistic understandings of behaviours among healthcare providers and managers in relation to data reporting. Recognising the performative (and counter-performative) nature of this activity, the paper demonstrates how calculative practices may be the arena for critical but obscured dependencies and power struggles that may paradoxically impede effective governance.

## Introduction

A focus on “transparent” metrics as objective, scientific indicators of good governance and accountability is common and growing in public sector organisations that are subject to competing demands and increasingly faced with pressure to emulate the private sector under new public management approaches (Muller, 2018; Mennicken and Espeland, 2019; McCabe, 2020).

The pursuit of transparency in healthcare systems internationally via rankings and indicators has intensified in the last 30 years (Mennicken and Espeland, 2019; Weber and Treem, 2024), where managing performance has focused on metrics such as waiting times, admissions to hospital and appointment numbers (Mannion and Braithwaite, 2012). This market-oriented drive offers the promise of transparency but may also give rise to unintended consequences, for example, failing to motivate service improvements through massaging figures to avoid penalisation – achieving the numbers while failing to examine underlying issues (Mannion and Braithwaite, 2012; Edwards and Black, 2023).

These outcomes point towards a range of resistant or sometimes subversive behaviours in the face of managerial interventions among those expected to comply with or enact them, a theme long explored in research on organisational change (Kotter and Schlesinger, 1979); as Dent and Goldberg (1999) note, people engage in resistance for multiple reasons, for example through fear of loss of status or control. This underlines the importance of considering the politics of resistance, which McCabe (2020) suggests has traditionally been neglected in this literature. Politics concerns individuals or groups engaging in power struggles to maintain or maximise their own interests. Power can be exercised variously, through conformity, opposition, cynicism and sabotage, and operates in a social relationship (McCabe, 2020).

### Metrics and reactivity in the public sector

While policy literature frequently seeks to increase transparency in healthcare systems, wider sociological disciplines, in particular critical accounting, argue that rankings, indicators and indexes are not static entities but have political effects on organisations and individuals (Hansen and Porter, 2012; Mennicken and Espeland, 2019; Soares and de Aquino, 2024). This literature posits that while the pursuit of transparency through such “calculative practices” is prized for its seeming ability to illuminate “dark corners” and offer reassurance to citizens and service users (Tweedie and Ronzani, 2024), such practices are never neutral devices and involve (overt or covert) power struggles and forms of resistance that can influence governance, making some things visible while obscuring others (Blomgren and Sunden, 2008; Boedker *et al.*, 2020). Thus, numbers not only describe but shape the world (Brorström, 2023). Such sociological studies show how human behaviour in public sector organisations changes in reaction to evaluation or observation in less predictable ways. Using the example of law school rankings, Espeland and Sauder (2007) highlight the concept of “reactivity effects”, where surveillance and/or measurement is interpreted in different ways by those observed, prompting unintended behavioural change. This can take the form of defensiveness and/or distrust when professionals may “lose discretion and … play to the test” (p. 2), focusing on indicators themselves to the exclusion of the qualities the indicators are intended to evaluate. In this way, rankings can produce resistance that creates “counter-performativity”, in which effects are the opposite of what is intended, for example, by paradoxically decreasing an organisation’s ranking results (Boedker *et al.*, 2020) or discouraging desired behaviours (Weber and Treem, 2024). Actors may also engage in micropolitical strategies to “game” performance metrics (i.e. bend but not break rules) to create the appearance of improved performance (Woelert, 2021).

### Metrics and reactivity in the healthcare sector

In the healthcare sector, several studies have analysed resistance to data scrutiny and ways in which actors seek (and manage) to avoid its “undesirable” consequences. For example, Exworthy *et al.* (2019) show how cardiac surgeons in the English National Health Service (NHS) hospital system acquiesced in managerial surveillance by complying with public reporting of patient mortality rates but controlling the data’s form and purpose, thus maintaining professional autonomy. Similarly, English NHS doctors gave a “tick-box” impression of conformity to new appraisal mechanisms while continuing with traditional practice (McGivern and Ferlie, 2007). Doolin (2004) highlighted the potentially double-edged nature of one calculative practice – a clinical resource usage monitoring system in a New Zealand hospital – which had financial implications for clinicians. Here, doctors complied superficially with data reporting but prevented managerial use of data by strongly disputing its accuracy, thereby avoiding behavioural control. More concerningly, McGivern and Fischer (2012) showed how clinicians subject to regulatory transparency became anxious and preoccupied with avoiding practices that could draw the attention of regulators; paradoxically they were more likely to hide potential malpractice because of transparency efforts. A focus on politics thus may reveal the ways actors exercise their power to potentially undermine the purpose of monitoring/measuring and work against it (Boedker *et al.*, 2020).

Critical, relational approaches have examined power asymmetries under surveillance (McCabe, 2020), noting that actors do not have an equal voice or can exercise power equally. For example, a comparative study on the introduction of a new policy in a French hospital to measure case-mix in two clinical disciplines – cardiac surgery and cardiology – offers insight into how more or less powerful professionals may react to surveillance attempts (Kern *et al.*, 2018). In contrast to the English cardiac surgeons above (Exworthy *et al.*, 2019), the French surgeons, whose presence was crucial to the hospital remaining operational, refused to comply with the new monitoring system. Management had no option but to accept the negation of the practice by powerful clinicians. By contrast, the cardiologists, who depended on hospital funding and whose power by extension was more limited, complied with scrutiny of their case-mix. Notably, these less powerful clinicians demonstrated ostensive compliance while ensuring the new measures did not disrupt their usual business. This study highlights power differentials between professional groups, showing how the less powerful cardiologists engaged in a form of “symbolic” policy implementation in which they complied with “the letter” of the policy, if not with “the spirit”. A study of routine health data reporting in Southern Tanzanian hospital wards saw healthcare workers of relatively limited influence exercising “discretionary” power by creatively handling data to deflect top-down scrutiny and balance the demands of busyness, pressure and patient needs (Molenaar *et al.*, 2025). Thus, even in situations with significant power differentials, people may find creative ways to reassert power and resist surveillance (McCabe, 2020).

Additionally, critical relational approaches to the examination of resistance/reactivity posit that defensive behaviours are not confined to those being assessed and/or monitored but may also extend to those doing the assessing (McCabe, 2020). Thus the “gaming” of systems may lead to organisations “hitting the target and missing the point” (Bevan and Hood, 2006). While the true purpose of measuring and/or monitoring is undermined here, the impression of accountability that is publicly displayed may allow both parties in a calculative practice – the assessor and the assessed – to avoid open tensions. In this way, the observed may use ostensible compliance to avoid “the calculating gaze” (Doolin, 2004), but regulators too may turn a blind eye to measurement issues, tacitly tolerating micropolitics to avoid a struggle (Bevan and Hood, 2006; Woelert, 2021). Reactivity effects then extend to include manager and/or regulator efforts to defend against potential conflict with those they are measuring or regulating (McGivern and Ferlie, 2007).

### Attitudes to metrics in English general practice

General practitioners (GPs) in the English NHS are subject to metrics for several purposes, e.g. professional appraisal and/or revalidation, quality assurance and performance management (Levene *et al.*, 2016). Previous research has touched upon stakeholder attitudes to sharing data and identified potentially resistant attitudes among GPs, for example, in their depictions of revalidation and/or appraisal as a burdensome tick-box exercise (Dale *et al.*, 2016). Indications of resistance also emerge in relation to public release of care quality data, where GPs raised concerns about unfair practice comparisons, loss of autonomy, the motivations of assessors (Khan *et al.*, 2020; Marshall *et al.*, 2002) and the inducement to “game” results (McGivern and Fischer, 2012). From the “assessor” side, governance leads may anticipate GP resistance to releasing data but be more concerned about the potential for this scrutiny and data release to damage their relationships with GPs (Marshall *et al.*, 2002). Nonetheless, political dimensions of data reporting are largely neglected in this literature.

Workforce data reporting is a key measurement activity whereby general practices must externally release staffing details. Anecdotal reports of misgivings among GPs and concerns about these data being linked to potential performance management (Edwards, 2016; Kelley-Patterson *et al*., 2017) hint at latent tensions here. However, research has not previously focused on general practice workforce data sharing as a potential arena for political reactivity effects, and this is the focus of our article.

### The context and the case

GPs in England are frequently both clinicians and independent business owners as GP partners, employing their own staff with services commissioned and/or funded through clinical commissioning groups (CCGs), now integrated care systems (ICSs). In December 2024, 6,227 English general practices were serving an average of 10,233 patients each (NHS England, 2024). Most are now part of a primary care network, a small group of practices in a geography working together to maximise access to additional funding (Fisher *et al.*, 2019). Some practices work as large, multi-practice providers while others are single practice. Beech and Baird (2020) explain the English general practice context where GPs are commissioned and/or funded to provide general medical services to a patient population via several income streams with a large proportion of funding paid per patient. In GP partnerships, GPs hold a joint stake in the business (sometimes with other clinicians and/or managers), sharing liability for fulfilling contracts, resources (clinical and/or administrative staff and estates) and income generated. Most practice spending goes on directly employed staff, meaning GP partners are usually jointly and severally liable for financial risks and are concerned to maximise net practice income.

The contractual requirement for English GPs to provide data on their workforce composition is an example of public accounting. Since 2015, workforce data have been collected via the National Workforce Reporting System (monthly since October 2020) and regularly, publicly released by an external body, NHS England. In addition to the national system, a range of commercially available workforce data collection systems have been developed, e.g. Healthy London Partnership (HLP) Primary Care Workforce Calculator (Transformation Partners in Health and Care, 2022) and Apex Insight tool (PA Consulting Group and Edenbridge Health Limited, 2018). The interest in workforce data is explained by reporting guidance which argues this data is crucial for understanding fluctuations in capacity, shaping local and system workforce planning and informing national policy and/or investment decisions on new workforce supply and training (NHS Digital, 2021).

The GP workforce reporting policy in England is simultaneously driven and encumbered by several challenges in general practice and the wider NHS; specifically, extreme pressure on the workforce due to high service demand (Buchan *et al.*, 2017), declining GP numbers and the introduction of new non-medical role professionals to share workload (NHS England, 2019; NHS England and BMA, 2019). In the wider NHS, workforce planning is often hampered by poor data quality, making it difficult to anticipate what workforce is needed (Addicott *et al.*, 2015) or conduct systemic workforce planning as proposed in health policy initiatives (NHS Improvement, 2020).

National workforce returns require regular updating by each practice, and improvement in data completeness over time suggests compliance has increased. For example, in December 2024 only 1.7% of full-time equivalent staff data required estimation due to incompleteness, compared with 9% in September 2015 (NHS England, 2024). However, policymakers have expressed concerns about data quality (NHS Digital, 2020) and reported GP cynicism about workforce data reporting (Kelley-Patterson *et al.*, 2017) points towards latent tensions and potential resistance.

The purpose of this paper is to go beyond assumptions that transparency is objective and decontextualised in the reporting of numbers within healthcare to engage with neglected political and performative dimensions of data. We suggest that workforce data reporting activity in English general practice is a new “space of contestation where the effects of quantification operate” (Mennicken and Espeland, 2019, p. 228). Our interest is in analysing (1) forms of resistance to data reporting among GP providers; (2) how resistances are interpreted and addressed by managers (commissioners and policymakers) and (3) the unintended consequences of unarticulated political dimensions. Framing this case as an exemplar of a health service-related calculative practice, we examine the enactment of workforce data reporting between providers and managers, revealing defensive reactivity effects which reflect contested meanings, motivations and power struggles in this sector. We seek to prompt a more nuanced debate on workforce data transparency drawing on the political dimensions of resistance to data reporting, with potential implications for wider healthcare and public sector policy, particularly in scenarios of external service commissioning.

## Methods

This research emerged from two studies: Study 1 examined the changing context of the general practice workforce in an English metropolitan region comprising 10 geographical areas; Study 2 focused on views of general practice workforce data reporting to inform a data collection tool for one area in the region. Synergistic elements between the studies – personnel, research focus and data interpretation – enabled these studies to be combined to generate insights on political tensions influencing data reporting and/or use, inspiring an integrated analysis.

We adopted a qualitative approach, using individual interviews (Study 1) and focus groups (Study 2) to gather a range of stakeholder views. Leaders with knowledge, expertise and strategic involvement in general practice workforce issues from provider (e.g. PCNs) or policymaker and/or commissioner organisations (e.g. NHS England and CCGs) were eligible for inclusion. Geographically, Study 1 sampled participants purposively at national, pan-regional and regional level (i.e. across all 10 regional areas); Study 2 at local level (one regional area). Both studies aimed to recruit GPs and/or non-GPs and snowball sampling supplemented recruitment. Ethical approval was obtained from the University Research Ethics Committee under “low risk” review as participants would be senior professionals (2017–2619-4613 and 2019–6731-10134).

Topic guides for each study tailored to distinct aims were developed from workforce literature, which, however, included common questions on workforce data reporting. Study 1’s guide focused broadly on workforce changes in the general practice context; Study 2’s focused more narrowly on workforce data tools and/or planning. Both guides, however, aimed to generate reflections on opportunities and/or challenges in workforce data reporting and ways to address them. Guides did not include direct questions about resistance to policy. Participant information sheets and/or consent forms were emailed to contacts invited to semi-structured interviews or focus groups, face-to-face at their workplaces or by telephone for their convenience. Researchers followed up invitations no more than twice, giving invitees the opportunity to ask questions, explaining they could skip any questions or stop and/or withdraw from the study at any time without giving a reason. Written consent was obtained from all invitees prior to participation. Data collection took place between August 2018 and October 2019 by a mixed-discipline team of non-clinical researchers, from health services research, medical sociology, management and organisational studies. All researchers, part of a regional university–NHS research collaboration, were experienced in researching the general practice and/or policy setting; this may have enhanced team credibility, enabled access to the participants (all in senior roles) and enabled them to regard us as peers. To further understanding of context and/or data interpretation, fieldnotes recorded details of the setting, researcher reflections and tentative analytic thoughts. Interviews lasted between 35 and 104 min; focus groups between 61 and 89 min. All were audio-recorded, transcribed verbatim, anonymised, held on a secure server accessible only to the researchers and organised in NVivo 12 software (QSR International Pty Ltd, 2018).

We used template analysis (TeA) as a broad approach to thematic data analysis widely applied in organisational and management research enabling generation of both pre-determined and emergent ideas (King and Brooks, 2018). Transcripts were read closely and coded in separate study databases; codes on data reporting were generated in both and considered jointly for similarities and/or differences. Initial insights on power relations between stakeholders were apparent across datasets – for example, mentions of reservations about data reporting, going through the motions and trust. Field notes helped draw further links between the study datasets. To help us refine analysis and/or interpretation of the combined data and integrate their synthesis in a methodologically consistent way, we used insights from the literature on quantification, transparency and reactivity effects as sensitising concepts. This literature served as a lens through which to consider power relations between service providers and managers in relation to data reporting across datasets. TeA enabled us to define higher order themes and conceptual relationships between them to reach an integrated, structured interpretation which illuminated forms of resistance to data reporting and responses.

Across both studies involving 32 interviews and four focus groups, our sampling strategy yielded 56 participants (27 GP providers and 29 non-GP policymaker and/or commissioners) at national, pan-regional, regional (all 10 areas of the region represented) and local levels (Table 1).

**Table 1:** Final study sample: national, pan-regional, regional and local provider/manager participants

Participant role	Number of participants in Study 1 interviews	Number of participants in Study 2 focus groups	Total participants Studies 1 and 2
*National (across England)*			
National GP provider leads	2		
National policymaker leads	4		
*Pan-regional (across one metropolitan region)*			
Pan-regional GP provider leads	6		
Pan-regional policymaker leads	2		
*Regional (across 10 areas of one metropolitan region)*			
Regional GP provider leads	12		
Regional commissioning leads	14		
*Sub-total*	*40 (17 GPs; 23 non-GPs)*		
*Local (within one area of metropolitan region)*			
Local provider clinical directors		5	
Local commissioning leads		5	
Local neighbourhood primary care leads		2	
Local practice managers		4	
*Sub-total*		*16 (10 GPs; 6 non-GPs)*	
*Grand total*			*56 (27 GPs; 29 non-GPs)*

**Source(s):** Authors’ own work

## Findings

Workforce data reporting was described as “*thorny*” by managers and “*difficult territory*” by GP providers, signalling felt but hidden tensions at the heart of this calculative practice. Our analysis identifies three distinct provider resistances to data sharing described by providers and managers alike – “surface”, “deeper” and “root” –and illuminates how managers interpreted and/or responded to each. Under this framing, we suggest that managers frequently misdiagnosed (and overlooked) political dimensions of data reporting, meaning proposed solutions to encourage data transparency failed to adequately address root resistance.

### “Surface” resistance: blaming technical/rational issues

Provider staff expressed negative views of workforce data reporting and blamed data tools for reduced engagement in a “surface” form of resistance, focused upon technical and/or rational problems. The national data tool was described as “*laborious*” (Provider Lead, Area 2), difficult to complete and “*clunky*” (CCG Lead, Area 8). Providers complained about duplication of effort in completing both national and local returns. Most regional areas had conducted local workforce data mapping with good response rates, achieved by heralding the activity as a one-off, leaving little opportunity for repeat exercises:

I think practices would get cheesed off if we did it annually … it’s duplicating things, certainly. (Provider Lead, Area 6)

Data tools were complex, but insufficiently sensitive to the nuances of the workforce, collecting partial data that overlooked variation in GP and/or practice nurse activity. These issues were compounded by the “*rapidly changing landscape*” of general practice (National GP Lead 1), meaning data became quickly obsolete. Notably, these technical and/or rational barriers did not prevent compliance; rather, providers complained but returned data nonetheless:

[Tool] will ask you if you’ve got nurses, and I’ve got loads … but they don’t do the same job! [And] because it’s generic – I put generic answers. So, it’s just not applicable. (Provider Lead, Area 2)

Provider staff conceded that “*garbage in*” meant “*garbage out*” (Practice Manager 4, Area 2), disclosing that returns were often completed hastily to “satisfice”:

I do a quick return and it’s not something I pay a great deal of attention to … it’s just a box-ticking exercise. (Practice Manager4, Area 2)

Further, providers admitted that going through the motions in this way would not help with workforce planning while resigned to mechanical (and by their own admission meaningless)data recording:

I end up just putting a number in. And I think to myself, well, that is totally useless. If anybody’s planning anything with that, they might as well give up now, because I couldn’t plan and I [unlike others] know what I’ve got! (Provider Lead, Area 2)

While providers complained about the practical and/or technical obstacles associated with data tools (the “surface” layer of resistance), they cursorily maintained compliance with the process. Some commissioners identified these rational problems as the main source of resistance, proposing that glitches could be overcome by simplifying the reporting process:

[We] overestimate the capability and knowledge within practices to [use data tools] … often there’s very little support … they’ve got a 45-page guidance booklet, it requires three different log-ins … the other way would be, you’ve just made it so simple and so easy that they’ve done it … (CCG Lead 3, Area5 )

However, this solution (reducing complexity and resolving technical problems) focused on addressing “surface” resistance alone, failing to attend to deeper issues in providers’ underlying attitudes and tokenistic behaviour.

### “Deeper” resistance: questioning the benefit/purpose

Some providers went further in doubting the benefits of data reporting and its fundamental purpose – a “deeper” form of resistance. From this view, releasing data would not enable providers to judge government workforce commitments:

We don’t really see how [workforce return] connects into the bigger picture … we don’t get it coming back to us in terms of how it has informed … the GP Five-Year Forward View or the Ten-Year Plan. We just assume it does, but you don’t actually know it does. (Practice Manager 3, Area 2)

Although the national return was a “mandatory” contractual requirement, completion did not link to tangible financial benefits, as sharing data did not generate direct practice income. This reduced provider inclination to fully engage:

It should be like everybody else’s contract everywhere else – you get money off the NHS – one of the expectations is you give us information back. It’s as simple as that for me. (Provider Lead, Area 7)

More broadly, it was argued workforce reporting did not add value to practices’ longer-term business strategy:

I can’t see a benefit to me on the ground of doing it … what do I get from it? I only want to spend time on things that are going to help me with resilience or business continuity … if it questioned me and made me think about how other practices do it but … it doesn’t tell me anything. (Practice Manager 1, Area 2)

Providers thus questioned the benefit of data reporting, and some openly queried its fundamental purpose. This suspicion was in part attributed to managers’ failure to articulate the reasons for data requests or give feedback; this “one-way” information sharing was said to underlie providers’ wariness:

Nobody starts with the “why” … [so] practices … can’t really see a connection between telling [reporting] they’re running on this number of GPs and practice nurses, with anyone actually being able to help them … so they don’t see … that connection at all and … I don’t think it’s been really attempted to articulate [it]. (Pan-regional GP Lead 6)

It’s one of those things that just sits in a black hole … I kind of know that as long as there is a tick in the box next to the practice’s name, most of the time [NHS England], are not going to look at the detail. (Practice Manager 1, Area 2)

Some managers thus believed the absence of a clear *quid pro quo* for practices was the main driver of resistant attitudes. The solution to this resistance would be to ensure practices “*physically get something back*” (CCG Lead 2, Area 5), such as feedback on returns to leverage practice income and “*all of a sudden they will find a way*” (CCG Lead 3, Area 5):

If people think we’re collecting all this data, and it goes into a big black hole … people get well and truly naffed off [annoyed]. So there has to be some feedback about value and it has to fight the corner for primary care as well, for a resource. (Neighbourhood Lead 2, Area 1)

In questioning the benefits and fundamental purpose of transparent workforce data reporting, providers edged towards articulating a “deeper” form of resistance to measurement. This points towards underlying issues of suspicion and mistrust of managers’ motives; however, by inferring that solutions lay only in providing feedback or incentivising the reporting process, managers again failed to adequately diagnose and address this second form of resistance.

### “Root” resistance: contesting the calculative practice

Concerns voiced about the “actual” purpose of workforce data requests signalled a third, more political tension in data reporting, centring on the relationship between stakeholders and arising from unclear and even hidden agendas on both sides – “root” resistance.

Showing an awareness of underlying political tensions, some commissioners admitted that providers wished to keep commercial aspects “*behind closed doors*” (CCG Lead, Area 9), resisting transparent data sharing to defend business autonomy. Relinquishing baseline workforce data alongside activity and/or appointment data might enable calculations about relative productivity at practice level. This created nervousness and encouraged some providers to conceal figures, with the potential to impact care:

People are quite secretive sometimes about what they actually do, so even if we ask them to fill it in anonymously … sometimes people do not give us the full picture … and it doesn’t really capture what the need of that population is. (GP Provider Lead, Area 9)

One GP leader went further, sharing that to maintain practice profitability some GPs may minimise practice headcount to the detriment of patient care (payments to practices being largely on a per patient basis) and consequently wish to obscure this detail from outside scrutiny:

There’s [a] paradox in general practice that is only really known to GPs, which is … if you offer a really poor service with no staff, you get rich, but if you offer a really, really good service with lots of staff you don’t make very much money … I suspect there’s a lot of GPs out there that don’t want people to particularly see which side of the line they’re walking. (Pan-regional GP Lead 4)

GPs conceded that their dual identity as “*doctors and businessmen*” (GP Provider Lead, Area 3), led them to resist attempts at benchmarking, standardisation and/or disclosure of commercially sensitive “*profit-per-patient*” details that could enable peer comparison:

It’s true that some practices staff themselves better than others … what GPs don’t spend on staff, they take home in their pockets – although it’s very controversial for me to say that! I think there is a fear you will be benchmarked against another practice that’s providing a better service for patients and a better working life for staff because they employ more people. (Pan-regional GP Lead 6)

By contrast, commissioners claimed their motivation in collecting data was to improve practices’ workforce planning and capacity, hampered – they contested – by the “*small business mentality*” (CCG Lead, Area 6) that drove some to resist complete data sharing. Policymakers felt this prevented the system obtaining a “*strategic workforce* *[…]* *overview*” (Regional Policy Lead 1). Beyond identifying improvements in workforce planning as a general goal, managers did not articulate how data collected could or would be used for improvement. Some conceded this may be “*a bit Big Brother-ish*” (CCG Lead, Area 7), reflecting ethical and moral concerns that NHS bodies might fail to reveal how data was used and/or fail to use it meaningfully. While this vacuum did nothing to dispel practice concerns about disclosing commercially sensitive data, it led some GPs to fill the void with their own judgements. The inference was that while ostensibly in place to assist workforce planning/capacity, the “true” point of workforce data requests was as “*a performance management tool*” (GP Provider, Area 10), to observe and monitor practices. The hostility towards data reporting among some GPs manifested as a more embedded form of political resistance to a calculative practice that might “*be used against them*” (GP Provider, Area 8):

There’s a lot of organisational reluctance to release data, because … a lot of GPs are quite paranoid about these tools. And sometimes they are right … because some … have used the data to hammer them. (GP CCG Lead 1, Area 1)

Knowledge is power … that’s our bargaining tool with [commissioners] … so let’s not give it to them on a free plate. (Practice Manager 3, Area 2)

This provider reactivity was underpinned by a long-established fundamental “*distrust and fear*” (CCG Lead, Area 6) between practices and NHS England and/or CCG commissioners:

Everything goes back to trust … and in the past … we have held people to account, and so people think “oh … someone’s checking my homework, and somewhere along the line I’m going to get into trouble.” (National Policy Lead 3)

Addressing the lack of trust was said to be “*absolutely key*” (Provider Lead, Area 6). However, the detail of how this might be achieved was largely skirted by managers, meaning that the “elephant in the room” – the unspoken problem of power and/or politics – was undisturbed. Some policymakers focused instead on what they perceived as a hopeful softening of relations:

I would hope that some of the relationships have … matured and developed … Think about what it was like back in 2013 when things changed over, it wasn’t a particularly positive time, but it does feel like we’ve moved on a lot from then. (National Policy Lead 4)

Some commissioners proffered light-touch changes to increase trust – the introduction of “GP champions” to bring “*peer credibility*” (CCG Lead, Area 6) or a relaxed “*ten-minute chat*” to “*allay* *[…]* *fears*” (CCG Lead 3, Area 5). One GP leader articulated the entrenched issues of power and trust between stakeholders that underlay workforce data reporting and could undermine the practice:

I think there’s some games being played by [general practice], by professional leaders, and [policymakers] … Everybody says we need this data to do proper planning, but no one quite seems to make anything happen, and I suspect a lot of people don’t want to know the answer – [policymakers don’t] want the hard data about the extent of the problem, and even professional bodies who are lobbying often are slightly concerned that the data might not confirm some of the emotive messages they’re sending out. (National GP Lead 1)

A third, more engrained layer of “root” resistance thus influenced data reporting, in which low trust levels and hidden agendas largely explained why GPs sought to contest (through tokenistic compliance), a calculative practice that some believed was designed to scrutinise their business models. Managers optimistically assumed that light-touch strategies (engaging GP champions and having informal conversations) would be sufficient to dissolve resistance while avoiding the more challenging problem of addressing relational power and trust.

In summary, proposed solutions to different modes of provider resistance to workforce data sharing reflected assumptions about what underpinned these resistances. Managers’ solutions largely misdiagnosed reactivity effects, overlooking the political nature of providers’ concerns and addressing only “surface” and “deeper” levels of resistance, while neglecting the more fundamental “root” challenge of power and trust.

## Discussion

In this article, we examine the mandatory reporting of general practice workforce data in the English NHS as a new arena of contestation around a calculative practice, revealing defensive reactivity effects among service providers and managers. As an example of healthcare data reporting, our analysis points to the need for a more nuanced debate about workforce data transparency and the potentially counter-performative nature of the reporting process.

Our analysis of this case highlights that GP providers ostensibly complied with workforce data reporting while admitting to a tokenistic form of data completion attributed to a range of practical and/or rational barriers. These barriers – too busy, tools overly complicated, failing to understand the purpose of the exercise, no return for efforts – are encompassed in what we term “surface” and “deeper” forms of resistance. We further identify a more political “root” form of resistance, where practices sought to avert exposure and scrutiny of individual business models and pushed back against a potential form of monitoring and/or control by revealing only partial information. We highlight that managers’ solutions to these surface and deeper resistances typically failed to address the “root” resistance – GPs’ embedded mistrust of a calculative practice perceived as “surveilling” their business activity (Table 2). Indeed, such solutions frequently distracted from the political power struggle at play.

**Table 2:** Provider forms of resistance and manager interpretations/responses

GP provider forms of resistance	Definitions of resistances	Commissioner and policymaker interpretations of/responses to resistances
Surface	Blaming technical/rational issues for problematic experiences of the data sharing process	Simplify the reporting process/tool
Deeper	Questioning the purpose and benefit of data sharing	Provide feedback on data returned and/or offer financial/business benefits for data sharing
Root	Contesting the “calculative practice” perceived as a mode of surveillance	Engage light-touch strategies that avoid addressing power dynamics between actors

**Source(s):** Authors’ own work

Our analysis supports prior anecdotal reports of “cynicism” among GPs about workforce data sharing (Edwards, 2016; Kelley-Patterson *et al.*, 2017) but goes further in highlighting the failure to recognise the performativity (and counter-performativity) in the process. We propose that the formal policy here, framed within a rationalist narrative of neutrality and transparency, reflects a misapprehension about the political dimension of resistance or, arguably, a reluctance to articulate and engage with root causes. Our analysis suggests that in some cases, managers circumvented these issues by “looking the other way” to avoid threatening their relationships with GPs as business owners.

Insights from critical literature on transparency, calculative practices, resistance and reactivity effects inform our understanding and interpretation of the case. We draw upon literature on the performative nature of reporting beyond English general practice to scenarios involving regulatory activities in the English NHS, the health services of different countries and international governance outside the healthcare field. We address three points pertinent to our case to raise questions about workforce data transparency and the potential for counter-performativity: the value of political understandings of surveillance and reactivity effects in data sharing practices and the influence of power dynamics on the defensive reactivity of providers and on commissioners and/or policymakers.

### Political understandings of surveillance and reactivity effects in data reporting

We argue firstly that political understandings of surveillance and/or data sharing behaviours are valuable, in that apparent compliance can mask resistance and/or subversion (Woelert, 2021; Molenaar *et al.*, 2025). Collecting and reporting numbers on health services in the name of transparency seeks to lay open professionals and organisations to accountability – ostensibly a rational and therefore “good” motive (McGivern and Fischer, 2012). Regulatory authorities hope or assume that capturing objective information leads naturally to positive reactivity effects, i.e. rational decision-making and professionals learning to review and “discipline” their own behaviour (Doolin, 2004). Framed thus, obstacles to data transparency are typically seen as largely technical in nature, to be rectified through “rationally” simplifying reporting processes or clarifying the benefits of sharing data. However, these interpretations underestimate the influence of power relations in transparency drives and the potential for defensive reactivity, where those being evaluated may resist managerial control through “mock rituals” (McGivern and Ferlie, 2007). Clinicians ostensibly complying with forms of surveillance while maintaining professional autonomy (e.g. doctors sharing performance data but controlling its form and/or use (Doolin, 2004; Exworthy *et al.*, 2019)) and clinicians’ “tick-box” compliance with appraisal mechanisms (McGivern and Ferlie, 2007) point towards a “transparency paradox” where actors find ways to avoid the undesirable consequences of actual transparency in data sharing (Weber and Treem, 2024).

In our case, far from enabling “positive” reactivity effects, such as prompting meaningful reflection and improved governance, the general practice workforce data sharing exercise saw GP providers complaining about seemingly rational obstacles while complying derisively and in a tokenistic manner – “symbolic” compliance. Further, the managers in our study focused on technical and/or rational interpretations of and solutions to the more “superficial” data problems raised by providers; however, using a calculative practices lens, we view these to lack a political interpretation, which points to covert forms of evasion and resistance that may undermine data reporting and work against transparency (Boedker *et al*., 2020).

### Power dynamics and the reactivity of providers

Secondly, we build on our first point to suggest that this covert evasion may reflect a relatively weak power position of GPs given dependence on commissioners and/or policymakers for funding, while simultaneously desiring to maintain and maximise independence. This is reminiscent of other scenarios where clinicians with limited influence have acted creatively to support their own interests in the face of managerial surveillance. For example, less powerful doctors dependent on hospital funding improved their unit’s performance by moving patients elsewhere (Kern *et al.*, 2018) and healthcare assistants making pragmatic trade-offs in health data reporting to avoid scrutiny (Molenaar *et al.*, 2025). In our case, GP autonomy has been long defined as a collection of social, economic and clinical freedoms (Schulz and Harrison, 1986). While a managerial-vs-professional and/or clinical distinction in autonomy dominates the literature, it is worth noting that this neglects the commercial tensions we highlight. Some GPs experienced workforce data sharing as a threat to their economic freedom, with defensive reactivity effects manifesting, at best, through tokenistic (and likely, low quality) data reporting, a “micro political” strategy which bent but did not break rules (Woelert, 2021). At worst, GPs returned a form of workforce data sufficient to fulfil regulatory obligations, while obscuring from outside scrutiny business models that may facilitate a low-cost, high-profit skeleton staffing structure. Worryingly, some informants suggested such behaviour may have indirect but negative effects on service quality and patient care. As English GPs are partially dependent on finance from NHS England and ICSs (formerly CCGs), this may reflect their weaker power position relative to other medical professions, resulting in a need to demonstrate compliance with demands while balancing their own interests with those of managers (Molenaar *et al.*, 2025).

#### Power dynamics and the reactivity of managers

Thirdly, we suggest that commissioners and/or policymakers may tacitly accept providers’ covert resistance to workforce data sharing to avoid surfacing tensions, resonating with prior observations that “hitting the target” can be a micropolitical strategy used by those under scrutiny, enabling assessors to circumvent open struggles (Bevan and Hood, 2006; Woelert, 2021). Thus, reactivity effects involving manager efforts to defend against potential conflict with those they are measuring seen elsewhere (McGivern and Ferlie, 2007) were also evident in our study.

The “gaming” effect seen in our case appeared to be exacerbated by a general reluctance between stakeholders to address the “elephant in the room” and articulate the underlying commercial tensions at the heart of general practice. The perception among our practices that workforce data provided went into a “black hole” may suggest that managers were not looking for measurement problems or indeed actively making meaningful use of data, as long as GPs publicly demonstrated their compliance with the mandate. While this permitted managers to avoid jeopardising relationships with GPs as independent business owners, it may also have enabled them to avoid full engagement with addressing the extent of workforce pressures. Thus, reactivity effects associated with this calculative practice led to a seeming level of complicity between stakeholders.

## Implications of the study

While the collection and reporting of data in healthcare and other arenas is typically seen as essential for transparency, good governance and informed policymaking, it also represents a powerful calculative practice, which is often the arena for obscured power struggles (Mennicken and Espeland, 2019). In this article, we have explored tensions and/or sources of resistance in the mandatory reporting of general practice workforce data in England. General practice faces a continuing decline in GP numbers and low morale, exacerbated by the COVID-19 pandemic. GP providers are vocal about workforce pressures while commissioners and/or policymakers present poor quality workforce data as a substantial obstacle to addressing such pressures. However, our study suggests that hidden motivations underlie the data reporting process. Building a common understanding of workforce issues based on reliable data will require a more open discussion between stakeholders in the commissioning-delivery scenario, which more realistically articulates political and/or power dimensions (McCabe, 2020).

These insights have wider application to international regulation and governance policy both within and outside healthcare. The literature cautions that calculative practices can be ineffectual exercises that paradoxically may hamper effective governance (Exworthy *et al.*, 2019; Weber and Treem, 2024) and may even hide poor practice (McGivern and Fischer, 2012). While metrics are not inherently undesirable, “metric fixation” in the absence of contextual knowledge does not advance our understanding of the complexity of what is really happening within organisations (Muller, 2018; Soares and de Aquino, 2024). Measurement activities require judgement, active management, the involvement of stakeholders in co-designing measures (Anders, 2024) and more open discussion of key questions, e.g. What kind of information is being measured and at what cost? How useful is the data and to whom? For what purposes will it be put (Muller, 2018)? Such “soft” intelligence from dialogue with and between stakeholders could go beyond “hard” metrics to offer richer insights into organisational problems, particularly when it highlights points of difference not only agreement (Mannion and Braithwaite, 2012).

If policymaking continues its focus on the pursuit of objective data, important underlying issues will remain unseen, creating greater misinterpretation, less understanding and less trust (Muller, 2018), defeating policy objectives and failing to “shape people’s aspirations” (Espeland and Sauder, 2007). Policymaking must therefore widen its focus from the neutral technical language of numbers to encompass power dynamics in governance approaches.

## Limitations and strengths

A limitation of this study is the distinctiveness of the semi-private nature of English general practice, which makes it more complex than other forms of healthcare provision, which may be state funded. However, this indicates that defensive reactivity effects associated with calculative practices may stem from a desire for financial independence and a reluctance to have business models scrutinised, as well as a concern for clinical autonomy. While the political sensitivities involved (GPs’ partial financial dependence on policymakers and/or commissioners and the latter’s apparent reluctance to jeopardise relationships with GP business owners) may have caused participants to be guarded, no participant skipped and/or appeared uncomfortable with questions and resistant attitudes and/or behaviours were widely disclosed, suggesting participants were not overly inhibited in their responses. Nor did the data suggest a perceived hierarchical relationship between stakeholders relative to each other. While the English hybrid model might present more space for resistance than in fully public systems, it does not alter the fundamental dialectic of reactivity to surveillance evidenced in the literature. Thus, the rich insights generated have broad transferability to wider data reporting policy in the healthcare and/or public sector, particularly in scenarios of external service commissioning.

## Conclusion

With reference to the wider international literature, we show how implementation of English general practice workforce data sharing – an example of calculative practice – raises questions about data transparency and the potentially counter-performative nature of the reporting process. In particular, the practice is hampered by managers’ misdiagnosis of the multi-faceted nature of provider resistances and the reluctance of stakeholders to openly address more political issues of commercial sensitivity and/or workforce pressures in this context. These insights have transferability to wider contexts of regulation and governance policy. Data collection and reporting in healthcare contexts (and beyond) is typically seen as essential for informed policymaking but also represents a powerful calculative practice, which results in unintended reactivity effects and masks power dynamics. This can result in fruitless, counter-performative data exercises that paradoxically derail attempts at transparency and even hide poor practice. We point to the politics inherent in “neutral” data reporting (Espeland and Sauder, 2007; Hansen and Porter, 2012) and the need to acknowledge and articulate contested meanings and motivations before they can be addressed.
